# Fever-related ataxia: a case report of CAPOS syndrome

**DOI:** 10.1186/s40673-019-0096-3

**Published:** 2019-02-08

**Authors:** Ida Stenshorne, Magnhild Rasmussen, Panagiotis Salvanos, Chantal M. E. Tallaksen, Laurence A. Bindoff, Jeanette Koht

**Affiliations:** 1 Department of Pediatric and Adolescent Medicine, Drammen Hospital, Vestre Viken Health Trust, Drammen, Norway; 2 Institute of Clinical Medicine, University of Oslo, Oslo, Norway; 3 Department of Clinical Neurosciences for Children, Oslo University Hospital, Oslo, Norway; 4 Department of Ophthalmology, Drammen Hospital, Vestre Viken Health Trust, Drammen, Norway; 5 Department of Neurology, Oslo University Hospital, Oslo, Norway; 6 Department of Clinical Medicine (K1), University of Bergen, Bergen, Norway; 7 Department of Neurology, Haukeland University Hospital, 5021 Bergen, Norway; 8 Department of Neurology, Drammen Hospital, Vestre Viken Health Trust, Drammen, Norway

**Keywords:** *ATP1A3* gene, CAPOS syndrome, Ataxia, Cerebellum

## Abstract

**Background:**

CAPOS (**C**erebellar ataxia, **A**reflexia, **P**es cavus, **O**ptic atrophy and **S**ensorineural hearing loss) syndrome is caused by the heterozygous mutation, c.2452G > A, in the *ATP1A3* gene. Other mutations in this gene can cause a spectrum of overlapping phenotypes including alternating hemiplegia of childhood, rapid onset dystonia parkinsonism, early infantile epileptic encephalopathy and fever induced paroxysmal weakness and encephalopathy. The phenotype is still mistaken for mitochondrial/metabolic disorders and follow up studies are scare.

**Case presentation:**

We report a 20 year old Norwegian male with ataxia, sensorineural deafness and visual loss. Before the age of five he experienced three fever related episodes of acute neurological deterioration when he temporarily lost his acquired motor skills and developed persistent gait and limb ataxia. In childhood, he developed bilateral optic atrophy and bilateral sensorineural hearing loss. Motor skills improved and at age 20 the patient showed a mild ataxia, hearing loss and reduced vision. A c.2452G > A mutation in the *ATP1A3* gene was identified and CAPOS syndrome was confirmed.

**Conclusions:**

This is the first Norwegian patient reported with CAPOS syndrome. Our patient had a de novo*,* previously identified *ATP1A3* mutation. The combination of recurrent episodes of fever related ataxia, loss of motor skills in early childhood, and early onset hearing and vision loss is typical of CAPOS syndrome. Previous reports suggest a gradual progression of the disease after the initial episodes, while this patient showed a good outcome with improvement of motor skills from adolescence long after the last deterioration episode.

**Electronic supplementary material:**

The online version of this article (10.1186/s40673-019-0096-3) contains supplementary material, which is available to authorized users.

## Background

CAPOS syndrome is an acronym for Cerebellar ataxia, Areflexia, Pes cavus, Optic atrophy, and **S**ensorineural hearing loss (OMIM; 601,338) and was first described in three patients from a family published by Nicolaides et al. in 1996 [1]. Since then, more than 40 patients have been reported [2–6]. The age of onset is between 6 months and 7 years and the first symptoms are acute episodes of ataxic encephalopathy or weakness triggered by febrile illness [2, 7]. Almost all patients have experienced one to three episodes of acute neurological symptoms [2, 7, 8], which usually recovered over days to months. Most patients have had a variable degree of persisting ataxia and develop areflexia, bilateral optic atrophy and sensorineural hearing loss over a few years [2]. Pes cavus is reported in 30% of patients [2, 9] and neuroimaging with cerebral magnetic resonance imaging is reported as normal in nearly all patients [1, 2, 4–6, 8–11] Demos et al. found a novel disease causing mutation c.2452G > A (p.Glu818Lys) in the *ATP1A3* gene*,* and subsequently the same mutation in this gene has been found in all patients with the clinical diagnosis of CAPOS syndrome [2, 7]. The mutation is known to be inherited in an autosomal dominant manner [5, 7, 9] and appears to be fully penetrant [12].

The *ATP1A3* gene encodes the alfa3 subunit of the Na^+^/K^+^ -ATPase, an ion pump, responsible for restoring neuronal membrane potential after depolarization and for maintaining neuronal excitability [13]. The alfa3 subunit is widely expressed in tissues such as the optic nerve, various parts of the cochlea, cerebellar cortex and nerves innervating muscle spindles [14–18]. Demos et al. hypothesized that the mechanism was consistent with a gain in function, and this has later been supported by Maas et al. [7, 8]. The defective ATPase appears to cause leakage of sodium and potassium ions across the cell membrane, which in turn leads to reduced neuronal excitability [19]. Animal studies have shown that in mice with a comparable defective ATPase, elevated temperature further increases the leakage of Na^+^/K^+^ ions [19], offering an explanation why fever may trigger the acute neurological signs in this syndrome [2, 8]. Other disease causing variants of the *ATP1A3* gene are known to cause: 1) Alternating hemiplegia of childhood (AHC); 2) Rapid onset dystonia parkinsonism (RDP); 3) Early infantile epileptic encephalopathy (EIEE); and 4) Fever induced paroxysmal weakness and encephalopathy (FIPWE) [12, 20–23]. The overlapping features make it difficult to differentiate these *ATP1A3*-related disorders clinically [20]. Fever seems to be the main trigger of the acute neurological symptoms in patients with CAPOS syndrome as well as FIPWE [2, 5, 23]. Other *ATP1A3-*related disorders have additional precipitating factors (i.e. illness, alcohol, physical or psychological events) [12, 21, 24].

## Case presentation

The following detailed presentation is based on the patient medical journal and supplemented by the parents’ written diary.

The patient was the first of two children of healthy, unrelated parents. He was born at term after an uncomplicated pregnancy and delivery. According to his parents, he reached all milestones at the expected times and showed a normal psychomotor development until the age of 8 months. The patient had three episodes of acute neurological symptoms requiring hospitalization at age 8 months, 18 months and 4 years. The symptoms and signs included reduced consciousness, general hypotonia, and ataxic movements that included problems with balance. The diagnostic workup was unremarkable in the acute setting, except for a slight elevation of C reactive protein (CRP) lending support to the diagnosis of a viral infection.

### Episode one at 8 months of age

The patient was admitted after a few days of febrile illness. He was dehydrated and weak with signs of opistotonus, but otherwise the examination was described as normal. He was treated with intravenous antibiotics for a suspected sepsis and his general condition rapidly improved. After three days he was discharged with a diagnosis of unspecific viral infection, and his parents cannot remember any motor problems after this hospitalization.

### Episode two at 18 months of age

The patient walked without support from the age of 14 months. At the age of 18 months, after a few days of being unwell with fever, acute laryngitis and conjunctivitis, he suddenly lost the ability to walk, had poor balance and uncoordinated extremities. The parents also described an episode two days before admission of reduced consciousness with additional abnormal body movements and hyperextension of his neck. His condition improved rapidly without specific treatment, and he was discharged with a diagnosis of nonspecific viral infection. Following discharge he had problems with motor skills: it took 2 weeks before he started to crawl, 4 weeks before he gradually started to walk, and 6 months until he could walk without support. His gait ataxia persisted after this episode.

### Age 2–4 years

Due to problems with balance and poor hearing, the patient was seen by a pediatric neurologist at age 2 years and 9 months. Examination was normal except of cerebellar deficits with a prominent trunk and gait ataxia, and therefore his gait was unstable and broad based and he was unable to jump. Deep tendon reflexes were absent. Electroencephalogram, blood tests, cerebrospinal fluid, as well as metabolic screening of blood and urine were normal. Cerebral magnetic resonance imaging (MRI) was also normal.

A diagnosis of bilateral secretory otitis was made and paracentesis was performed. His hearing and speech improved markedly after this procedure. The otoacustic emission was reported as normal. Ophthalmologic examination showed normal vision, but a mild horizontal nystagmus and bilateral optic atrophy. From the age of 3 years, the parents reported that the boy had impaired vision.

### Episode three at 4 years of age

The patient was again admitted to the hospital at the age of 4. He had been unwell for a few days with fever and an exanthema due to a possible childhood illness. On admission he was described as hypotonic and unable to stand, with ataxia in the trunk and limbs. His head control and visual fixation were poor and he gave no eye contact. His condition rapidly improved without specific treatment. Over the next 6 months the motor skills gradually improved, but the gait, trunk and limb ataxia persisted.

### The diagnostic odyssey (age 4–20 years)

In total, more than 100 consultations at different hospitals were performed. Repeated investigations with normal findings included nerve conductions, electromyography, cerebrospinal fluid, muscle biopsy, metabolic tests in blood and urine, MRI of the brain (at the age of 2, 4, 6 and 9 years), single gene tests (*TIMM8A,* Connexin 26, Connexin 30, frataxin, *ABHD1, POLG,* otofelin, *TL1, TK, ATP*), array-cgh, electroencephalograms and in addition different vision and hearing assessments.

At the age of 4 he was, in addition to the ataxia, diagnosed with bilateral auditory neuropathy due to a pathological auditory brainstem response. The diagnosis of Mohr-Tranebjærg syndrome was mentioned as a possible diagnosis, but was not genetically confirmed. From the age of 5 the ataxia has been reported to gradually improve.

At the age of 7 he was diagnosed with reduced vision. His best spectacle corrected visual acuity with the age appropriate test was 0.5 in both eyes with an obvious “crowding” effect, and the contrast sensitivity with the Hiding Heidi low contrast face test (Good Lite, IL, USA) was slightly reduced at 2,5%. The visual evoked potential and electroretinogram was at that time described as normal.

Throughout childhood he was also investigated and had different diagnoses due to concentration problems and learning difficulties, but his behavioral issues improved drastically at the age of ten, when he was taught sign language. As an adolescent the patient received cochlear implants bilaterally, and his school performances improved after this.

Due to the bilateral cochlear implants no later MRI investigations after the age of nine have been performed.

At the age of 16, the patient was reevaluated by an adult neurologist. At that time he was well functioning, but with a mild gait and limb ataxia and significant problems with hearing and vision due to known bilateral sensorineural hearing loss and optic atrophy. In addition he had a general areflexia.

### Examination and investigations at the age of 20 years

The patient is well functioning and has completed high school education. He still has a mild gait and limb ataxia, which worsen when walking on uneven surfaces, and he is not able to walk or stand in tandem (Additional file 1).


**Additional file 1:** Video of the patient illustrating the ataxia (MP4 40537 kb)


**Fig. 1:**
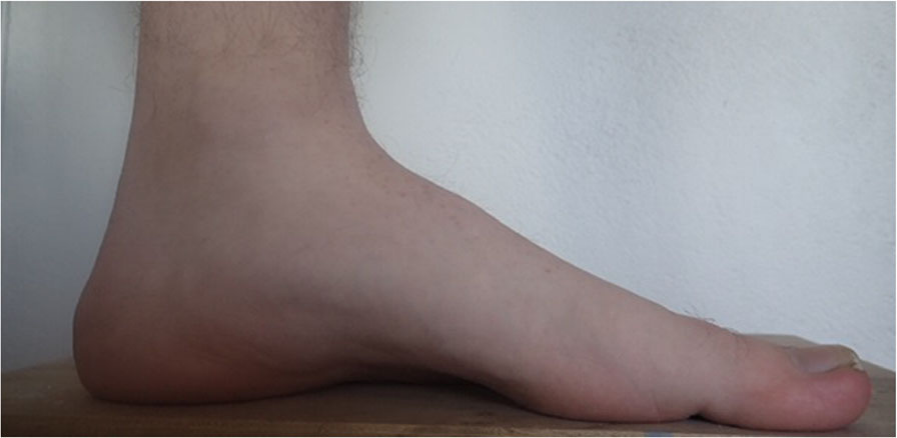
Left foot of the patient

**Fig. 2:**
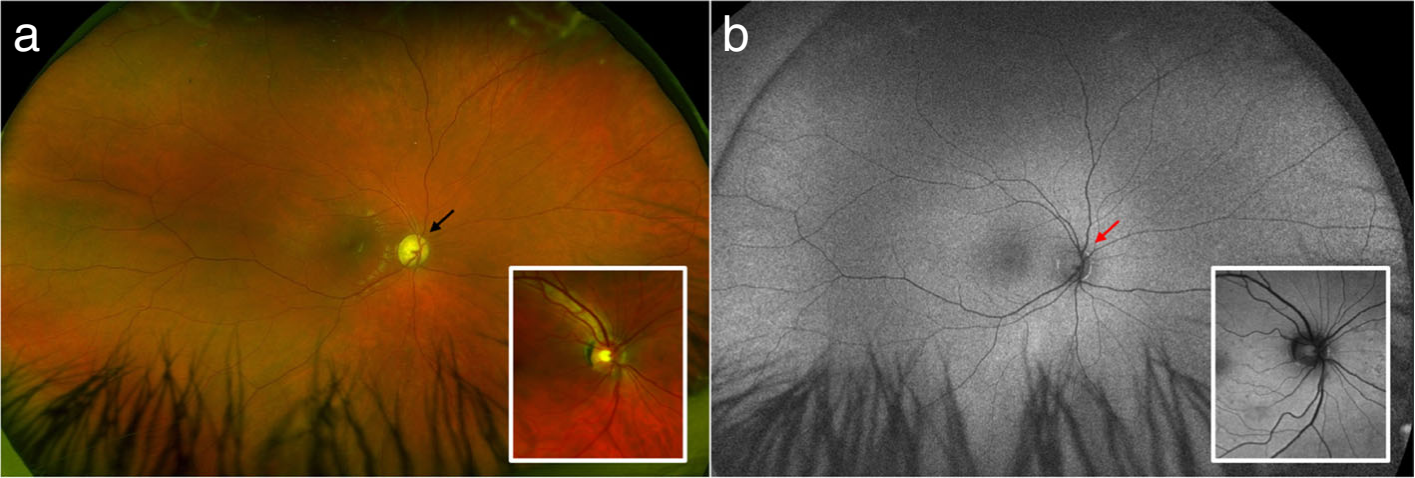
Ultra widefield retinal imaging of the right retina. **a** Ultra-widefield color fundus image of the patient´s right eye showing atrophy of the optic nerve head (black arrow). **b** Ultra-widefield autofluorescent fundus image of the patient´s right eye showing loss of the normal hypofluorescence (darker signal) of the optic nerve (red arrow) due to loss of nerve fibers that leads to window-defect and hyperfluorescence from the underlying sclera. The rest of the ocular fundus shows normal autofluorescent signal, indicating a normal retinal pigment epithelium and photoreceptors. The inserts show corresponding color and autofluorescent findings in a normal, age-matched individual. The images of the left eye were corresponding (not shown here)

He has a slight intention tremor and dysmetria, but no dysdiadochokinesis. Scale for the assessment and rating of ataxia (SARA score) is 10 out of 40 points. Muscle tone is normal, as is sensation, except for mildly impaired pain sensation distally. There is a general areflexia and plantar reflexes are down going. He has slightly high feet arches (Fig. 1), but no problems with his feet. He has bilateral cochlear implants and is able to speak, although speech is affected by his hearing impairment and he prefers to use sign language. Spectacle corrected visual acuity is 0.1 in both eyes, and he has gaze evoked horizontal nystagmus. Contrast sensitivity is reduced further to 5%. Slitlamp examination revealed no anomaly in the anterior segment, while dilated ophthalmoscopy shows bilateral diffuse optic atrophy. Ultra widefield fundus photography confirms the pale, atrophic optic nerves with otherwise normal retina and vessels. Autofluorescence imaging confirms the finding, showing loss of nerve fibers on the optic disc that in normal individuals give darker, hypofluorescent signal and revealing the hyperfluorescent sclera and lamina cribrosa (Fig. 2).

Diagnostic exome sequencing of the patient and his parents revealed a de novo mutation: c.2452G > A; p.Glu818Lys in the *ATP1A3* gene in the patient.

## Discussion and conclusions

This is the first patient reported with CAPOS syndrome in Norway. While previous reports have suggested gradual progression of disease [2], our patient showed improvement of the ataxia from the age of five until the age of 20 years. Our patient has four of the five main features of CAPOS syndrome. He has high feet arches, but no pes cavus. This is the most debated feature of the syndrome and is present in only 30% of patients as opposed to 10% of the general population [2, 9]. We would therefore support the suggestion that P should be omitted and the acronym changed to CAOS syndrome [9]. In addition to the well known features of CAPOS syndrome, our patient has bilateral horizontal nystagmus. Until recently, unspecified nystagmus is reported in over 50% of all CAPOS syndrome patients and many of them are specified as horizontal nystagmus [1, 2, 7–10, 25]. Nystagmus therefore seems to be a common feature of the syndrome. During the first and second acute episodes opistotonus, abnormal body movements and hyperextension of the neck were described. We believe this has been dystonia. No videos or more objective descriptions of these features were done in the medical record. Dystonia and other hyperkinetic movements are described in a few previously reported CAPOS syndrome patients [7, 8, 24], but are more commonly described in other *ATP1A3*-related disorders [26].

The specific *ATP1A3* gene mutation found in this patient underlines the fact that this genetic mutation specifically is confined to patients with CAPOS syndrome, and is not found in association with other *ATP1A3-*related syndromes [2].

This patient went to more than 100 consultations and underwent numerous investigations at different hospitals in the space of 20 years. Many of these investigations are expensive and many done repeatedly. Most test results have been unremarkable and did not give much guidance to the correct diagnosis. A long list of differential diagnoses has been evaluated, but no explanation for his signs and symptoms was found before a new clinical and genetic evaluation was done. Many of the reported CAPOS syndrome patients were initially suspected of having other diseases such as; parainfectious cerebellitis [8], encephalitis [2], metabolic disease [1, 8], mitochondrial disease [1, 2, 8, 9], episodic ataxia type 2 [8], Miller-Fisher syndrome [9] or atypical Guillain-Barré syndrome [8], dominant optic atrophy plus (OPA1) [2, 9], spinocerebellar ataxias [2], as well as many cerebellar ataxic syndromes [1]. For many years the persisting ataxia, hearing and vision loss in this patient were suspected to be caused by a metabolic or a mitochondrial disorder. CAPOS syndrome may resemble these disorders as they all can be associated with multiorgan affection as well as paroxysmal attacks of hypotonia, poor contact and poor general condition, typically occurring with intercurrent disease. Nevertheless, the positive clinical picture of our patient with gradual improvement of the ataxia did not fit well with a metabolic or a mitochondrial disorder where the natural course would be a progressive ataxia. The same applies to his normal test results that include metabolic screening, MRI of the brain and previous genetic workup. In the last decade there have been an increasing number of reports of CAPOS syndrome [2–6, 11].

Fever is the main trigger of acute neurological symptoms of CAPOS syndrome and FIPWE [2, 5, 23], and this is also seen in our patient. When investigating a young child with fever related ataxia *ATP1A3*-related disorders should therefore be considered.

As for most rare disorders, many diagnoses have been suspected and checked for in this patient, which is also the case for many of the other reported patients with CAPOS syndrome [1, 2, 8, 9]. There are more than 5200 reported monogenetic diseases in 2018 (http://omim.org/statistics/entry) and it is impossible to know all the phenotypes. Today we know that mutations in the *ATP1A3* gene are common in ataxia patients and should be tested for [27]. Monogenetic diseases is a difficult field in medicine, but with rapidly evolving molecular genetic diagnostic tools, as well as available and searchable databases, it has become possible to diagnose these patients today, and will probably be much easier in the future.
